# CpG-oligodeoxynucleotides developed for grouper toll-like receptor (TLR) 21s effectively activate mouse and human TLR9s mediated immune responses

**DOI:** 10.1038/s41598-017-17609-2

**Published:** 2017-12-11

**Authors:** Da-Wei Yeh, Chao-Yang Lai, Yi-Ling Liu, Chih-Hao Lu, Ping-Hui Tseng, Chiou-Hwa Yuh, Guann-Yi Yu, Shih-Jen Liu, Chih-Hsiang Leng, Tsung-Hsien Chuang

**Affiliations:** 1 Immunology Research Center, National Health Research Institutes, Miaoli, Taiwan; 2 Department of Life Sciences, National Central University, Taoyuan, Taiwan; 3 Institute of Biochemistry and Molecular Biology, National Yang-Ming University, Taipei, Taiwan; 4 Institute of Molecular and Genomic Medicine, National Health Research Institutes, Miaoli, Taiwan; 5 National Institute of Infectious Diseases and Vaccinology, National Health Research Institutes, Miaoli, Taiwan; 6 Program in Environmental and Occupational Medicine, Kaohsiung Medical University, Kaohsiung, Taiwan

## Abstract

Synthetic phosphorothiolate-modified CpG-oligodeoxynucleotides (CpG-ODNs) are potent immune stimuli. Toll-like receptor (TLR) 9 and TLR21 are their cellular receptors in different species. The structural requirements for CpG-ODN to strongly activate TLR9 have been relatively well studied, but studies on TLR21 are in their infancy. Therefore, in this study, we investigated the interaction between CpG-ODNs and TLR21s from groupers (*Epinephelus* spp.), which are economically important fish species. We cloned the cDNA of giant grouper (*E. lanceolatus*) TLR21, and compared its sequence with orange-spotted grouper (*E. coioides*) TLR21A and TLR21B. These three receptors were activated by CpG-ODNs containing the GTCGTT motif but not by those containing the GACGTT motif. We developed two CpG-ODNs that contained 19 phosphorothiolated deoxynucleotides with one or two GTCGTT motifs. These CpG-ODNs had better activity on grouper TLR21s than currently developed CpG-ODNs, and produced similar immune stimulatory profiles when applied to cells isolated from orange-spotted grouper. The developed CpG-ODNs also effectively activated both human and mouse TLR9-mediated NF-κB activation and cytokine productions. These findings suggest that the GTCGTT motif is required for CpG-ODNs to activate grouper TLR21s, and that the CpG-ODNs that were developed for grouper TLR21s contain structures that effectively activate human and mouse TLR9s.

## Introduction

Synthetic phosphorothioate-modified CpG-oligodeoxynucleotides (CpG-ODNs) are potent immune stimuli^1–4^. In mammals, they induce the production of inflammatory cytokines and a T helper 1 (Th1) polarized immune response, resulting in the expression of costimulatory molecules in antigen-presenting cells, and increased activation of B cells, T cells, and NK cells^5–8^. Similarly, in avian and fish species, they have been shown to stimulate cytokine production and induce the activation of different leukocytes^9–11^. Consequently, CpG-ODNs are currently been investigated for use as immune modulators and vaccine adjuvants in humans and various other species, including domestic animals, birds, and fish^12–18^.

In general, CpG-ODNs contain one or more copies of CpG-deoxynucleotides containing hexamer (CpG-hexamer) motifs, and their immunostimulatory activity depends on the number, position, spacing, and surrounding bases of these motifs^19–21^. CpG-ODNs also have species-specific activity, which is determined by their structural features, such as the context of the CpG-hexamer motifs and their length^8,22,23^. For example, CpG-1826, which contains two copies of the GACGTT motif and has a length of 20 nucleotides, is more potent in activating murine cells and less effective in activating human cells than CpG-2007, which contains three copies of the GTCGTT motif and has 22 nucleotides. In contrast, the CpG-2007 is more effective in activation of human cells than the CpG-1826^8,22–24^. In rabbit cells, CpG-C46 and CpG-C4609, which contain a GACGTT and AACGTT motif, respectively, and are 12 nucleotides long, generate stronger immune responses than CpG-1826 and CpG-2007^25^.

Toll-like receptors (TLRs) recognize pathogen-associated molecular patterns from microbes, leading to the initiation of host responses to microbial infections^26–31^. Ten TLRs (TLR1–10) have been identified in human cells and 13 TLRs (TLR1–13) have been identified across all mammalian species^32,33^. Several additional TLRs have also been identified in other vertebrate lineages, including TLR14, TLR15, and TLR18–27. In fish, around 20 different TLRs have been identified, including both mammalian and non-mammalian TLRs^11,34^. Mammalian TLR9, and non-mammalian TLR21 which has been identified in avian and fish species, are the cellular receptors for CpG-ODNs^35–38^. Most of our current knowledge about the structural–functional relationships and species-specific activities of CpG-ODNs is generated by studying their interaction with TLR9, but much less is derived from the study with TLR21. This is because most of the earlier work on CpG-ODNs was performed using human and mouse cells, which contain only TLR9. Moreover, TLR9 was identified earlier than TLR21. TLR21 was identified later in zebrafish (*Danio rerio*) and pufferfish (*Takifugu rubripes*) genomes, followed by other fish species and chicken (*Gallus gallus*)^35,39,40^. Previous studies have shown that chicken and zebrafish TLR21s are activated by CpG-ODNs^35,38,40^. However, the sequence that is required to strongly activate TLR21 has not been investigated and it remains unclear whether a CpG-ODN can simultaneously have strong activity toward both TLR9 and TLR21.

Many different species of groupers (*Epinephelus* spp.) inhabit tropical waters in the Indo-Pacific region, some of which have been farmed, with giant grouper (*E. lanceolatus*) possibly being the most economically valuable aquaculture species. Much like other farmed fish species, groupers are susceptible to viral and bacterial infections, and so effective immune modulators, including vaccines and vaccine adjuvants, are required for optimal aquaculture^12,41–43^. Therefore, in this study, we investigated the interaction between CpG-ODNs and grouper TLR21s, with the dual aim of gaining a better understanding of the structural basis of the interaction and helping to develop effective immune modulators for this species. To do this, we cloned giant grouper (gg) TLR21 cDNA and performed a cell-based activation assay with expression vectors for ggTLR21, and orange-spotted grouper (osg, *E. coioides*) TLR21A and osgTLR21B. This led to the development of CpG-ODNs that not only had stronger activities toward these grouper TLR21s, but also effectively activated human and mouse TLR9s, suggesting a similar structural requirement for the activation of TLR21s and TLR9s from different species.

## Results

### Molecular cloning of ggTLR21

The cDNA from osgTLR21A and osgTLR21B has previously been cloned^44^. The main difference between their encoded protein sequences is the absence of four amino acid residues at the C-terminal end of osgTLR21B (Supplementary Fig. 1). Based on the expected high identity between nucleotide sequences of the same gene in giant grouper and orange-spotted grouper, we designed two primers based on the 5′- and 3′-untranslated regions of osgTLR21 to clone ggTLR21. This resulted in a full-length ggTLR21 cDNA being cloned that was homologous to osgTLR21A, whereas the homologous to osgTLR21B was not identified in the giant grouper cDNA library. The protein identity was 98.26% between ggTLR21 and osgTLR21A, 98.15% between ggTLR21 and osgTLR21B, (Supplementary Fig. 1).

The GenBank database contained more protein sequences for different TLRs from zebrafish than any other fish species due to the zebrafish genome having been sequenced^45,46^. To compare the grouper TLR21s with other TLRs, we aligned their protein sequences with those of different zebrafish TLRs using ClustalW2 and constructed an evolution tree. TLR22 is closest to TLR21 in the evolution tree, with TLR20f and TLR13 the next closest (Supplementary Fig. 2). Further phylogenetic analysis using the protein sequences of TLR21 from different fish and avian species showed that the grouper TLR21s are most closely related to *Oplegnathus fasciatus* TLR21, and are more distantly related to chicken (*Gullas gullas*) and goose (*Anser cygnoides*) TLR21s. Moreover, besides the identification of two isoforms for osgTLR21, only the *Cyprinus-carpio*TLR21 was found to have two isoforms. (Supplementary Fig. 3).

### Protein sequence analysis of grouper TLR21s

**Figure 1:**
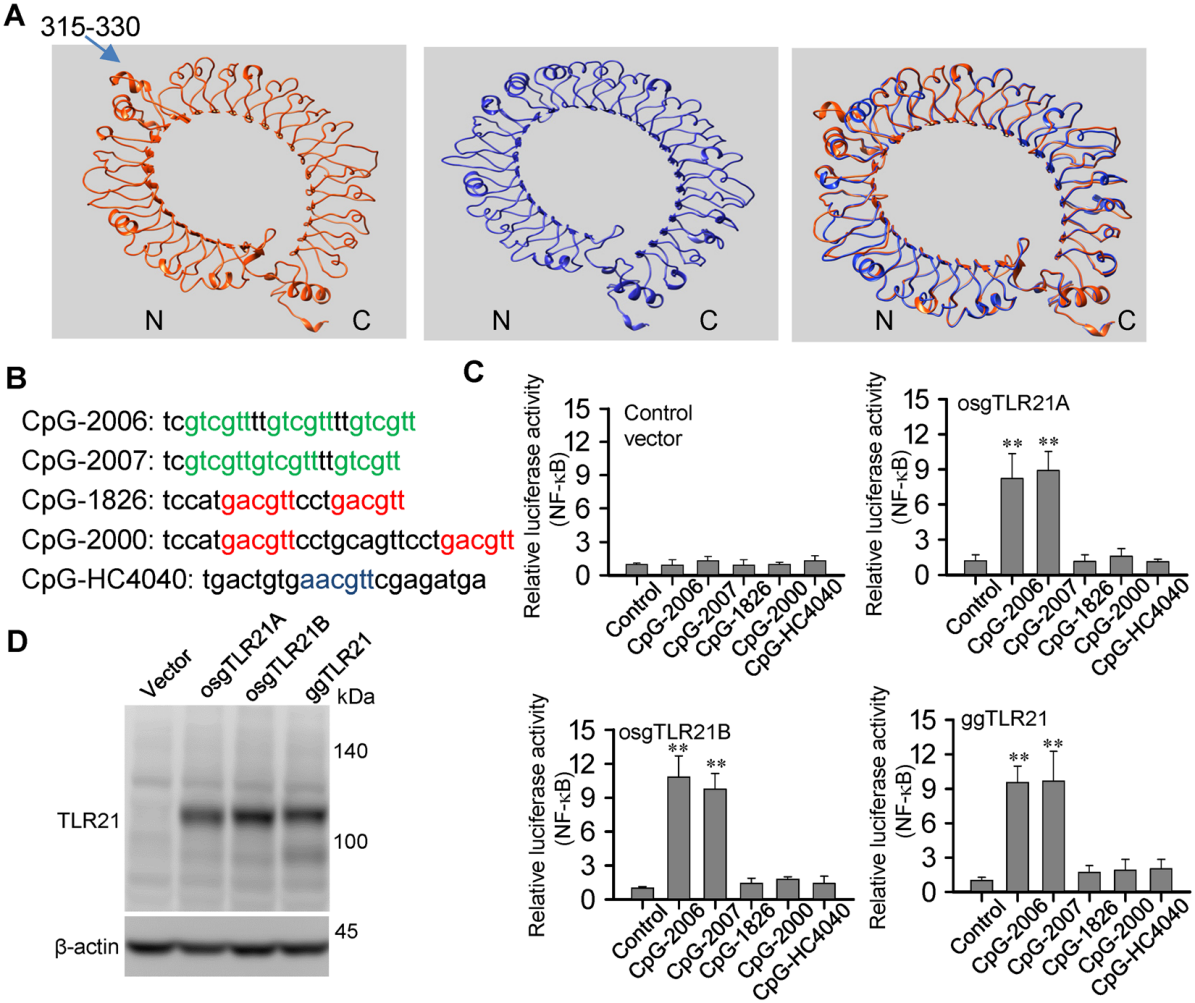
Ectodomain protein structures of grouper (*Epinephelus* spp.) toll-like receptor (TLR) 21s and activation of these TLRs by different CpG-oligodeoxynucleotides (CpG-ODNs). (**A**) Computational modeling of the ectodomain protein structures of orange-spotted grouper (osg, *E. coioides*) and giant grouper (gg, *E. lanceolatus*) TLR21s. From left to right: predicted ectodomain structure of osgTLR21, ggTLR21, and superimposition of these two ectodomains. N: N-terminal end, C: C-terminal end of the ectodomain. (**B**) Sequences of CpG-ODNs used in this study. (**C**) Relative luciferase activities of human embryonic kidney (HEK) 293 cells co-transfected with a control vector and expression vector for different grouper TLR21s as indicated, along with a nuclear factor (NF)-κB controlled luciferase reporter gene, and treated with 3 µM CpG-ODN for 7 h. Data represent means ± SD (n = 3 independent experiments). **P < 0.01 compared with the control. (**D**) Immunoblot analysis of the expression of the grouper TLR21s using β-actin as a loading control.

ggTLR21 contains 979 amino acid residues, while osgTLR21A and osgTLR21B contain 979 and 975, respectively. Alignment of these three protein sequences showed that they contain an extracellular domain (ectodomain), a transmembrane domain, and a Toll/IL-1 (TIR) cytosolic domain. They also have 23 copies of leucine-rich repeats (LRRs) and a C-terminal leucine-rich repeat (LRR-CT) in their ectodomain. Only 14 of 741 amino acid residues differed between the ectodomain of osgTLR21 and ggTLR21 (Supplementary Fig. 1). Several three-dimensional structures of different TLR ectodomains have previously been resolved^47,48^, of which TLR13 is phylogenetically closest to TLR21 (Supplemental Fig. 2). Therefore, we predicted the three-dimensional structures of the osgTLR21s and ggTLR21 ectodomains with SWISS MODEL software using TLR13 as a template to further examine their difference. This showed that their ectodomains have relatively similar horseshoe-shaped solenoid three-dimensional structures, except for the extrusion of a small helix structure at the 315–330 amino acid region in osgTLR21 (Fig. 1A). The three motifs (boxes 1–3) that are required for signal transduction of mammalian TLRs^49–51^ are conserved in the TIR domains of all three grouper TLR21s. However, osgTLR21B differs from osgTLR21A and ggTLR21 in that it lacks a four amino acid residue region at the C-terminal end after box 3 (Supplementary Fig. 1).

### Activation of grouper TLR21s by CpG-ODNs

To investigate whether the structural differences shown in Fig. 1A between these grouper TLR21s translates into differences in their activity in response to CpG-ODN stimulation, we employed cell-based TLR21 activation assays for studies. We treated the TLR21 expression vector and reporter gene co-transfected cells with several currently developed and frequently used CpG-ODNs with different hexamer motifs and sequences as shown in Fig. 1B, and measured the induced luciferase reporter activities. We found that osgTLR21A, osgTLR21B, and ggTLR21 experienced the same level of activation by CpG-ODNs containing GTCGTT hexamer motifs (i.e., CpG-2006 and CpG-2007), but did not respond to CpG-ODNs containing GACGTT and AACGTT hexamer motifs (i.e., CpG-1826, CpG-2000, and CpG-HC4040) (Fig. 1C and D). These findings suggest that all three grouper TLRs are functional and these minor structural differences do not significantly affect their response to CpG-ODN stimulation.

### Development of CpG-ODNs for strong activation of grouper TLR21s

**Figure 2:**
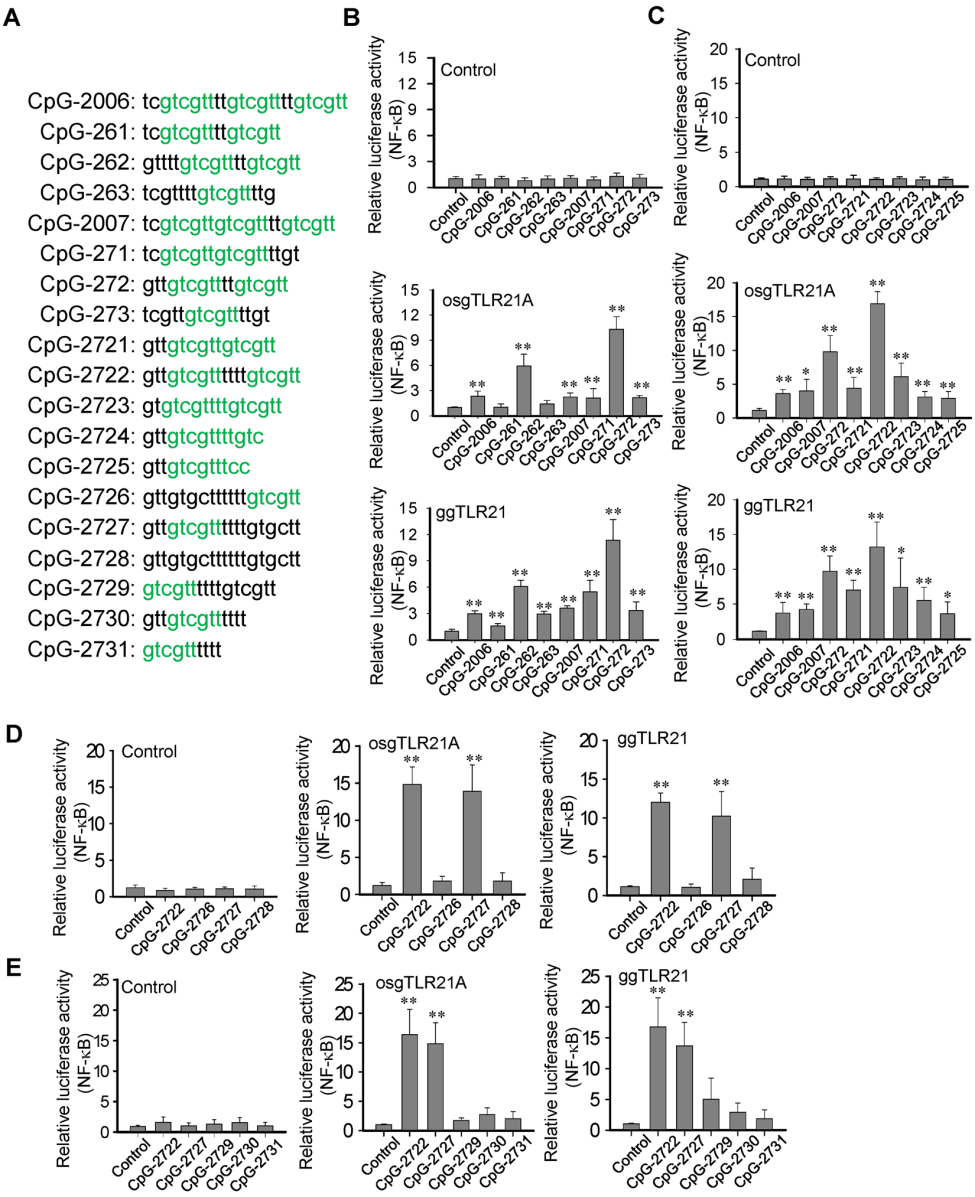
Development of CpG-oligodeoxynucleotides (CpG-ODNs) for strong activation of grouper (*Epinephelus* spp.) toll-like receptor (TLR) 21s. (**A**) Sequences of CpG-ODNs used in this study. (**B**–**E**) Relative luciferase activities of human embryonic kidney (HEK) 293 cells co-transfected with a control vector and expression vector for orange-spotted grouper (osg, *E. coioides*) and giant grouper (gg, *E. lanceolatus*) TLR21s as indicated, along with a nuclear factor (NF)-κB controlled luciferase reporter gene, and treated with 0.3 µM of CpG-ODNs derived from CpG-2006 and CpG-2007 (**B**), CpG-ODNs derived from CpG-272 (**C**), CpG-2722, −2726, −2727, −2728 (**D**), and CpG-2722, −2727, −2729, −2730, −2731 (**E**), for 7 h. Data represent means ± SD (n = 3 independent experiments). *P < 0.05, **P < 0.01 compared with the control.

Previous studies have indicated that in addition to the number, position, spacing, and surrounding bases of the CpG-hexamer motifs, the length of CpG-ODNs is also important in determining their immunostimulatory activity^25^. Therefore, in an attempt to develop CpG-ODNs for strong activation of grouper TLR21s, we trimmed the length of CpG-2006 and CpG-2007 to retain only the left or right two of the three GTCGTT hexamer motifs to generate CpG-261 and CpG-271, and CpG-262 and CpG-272, respectively (Fig. 2A). We also trimmed their length to retain only the middle copy of the hexamer motif to generate CpG-263 and CpG-273 (Fig. 2A). We then determined the activities of these CpG-ODNs using an osgTLR21A and ggTLR21 cell-based activation assay, and compared them with CpG-2006 and CpG-2007. We found that CpG-272 had the best activities toward the osgTLR21A and ggTLR21 (Fig. 2B). Therefore, we further modified CpG-272 by trimming its length or changing the spacing between the two hexamer motifs to generate CpG-2721 to CpG-2725 (Fig. 2A). A further cell-based assay indicated that CpG-2722 had the best activity toward the osgTLR21s and ggTLR21 (Fig. 2C).

### Number of CpG-hexamer motifs and structure required for the CpG-2722 to activation of grouper TLR21s

CpG-2722 contains two copies of the CTCGTT hexamer motif in a length of nineteen nucleotides. To determine whether both copies are required for its activity, we reversed the CpG-dideoxynucleotides in its 5′- and 3′-CpG-hexamer motif to generate CpG-2726 to CpG-2728 (Fig. 2A). The results from osgTLR21A and ggTLR21 activation assays indicated that the activity of CpG-2727 was as good as that of CpG-2722, whereas CpG-2726 and CpG-2728 were unable to activate the osgTLR21A and ggTLR21 (Fig. 2D). These findings suggest that only one copy of the 5′-CpG-hexamer motif is required for CpG-2722 activity. The CpG-2722 was further trimmed to generate CpG-2729 by removing three nucleotides from 5′-end, CpG-2730 by removing the 3′-CpG-hexamer motif, and CpG-2731 by removing both of the three nucleotides from 5′-end and the 3′-CpG-hexamer motif (Fig. 2A). The activities of these CpG-ODNs were tested. The results showed that the three 5′-end nucleotides were required for the CpG-2722 to strongly active both of the osgTLR21 and ggTLR21, and although the 3′-CpG-hexamer motif is not required for the activity of CpG-2722, these nucleotides are required to maintain the nineteen nucleotide length of CpG-2722 for its activity (Fig. 2E).

### Effect of grouper TLR21-activating CpG-ODNs on human and mouse TLR9s

**Figure 3:**
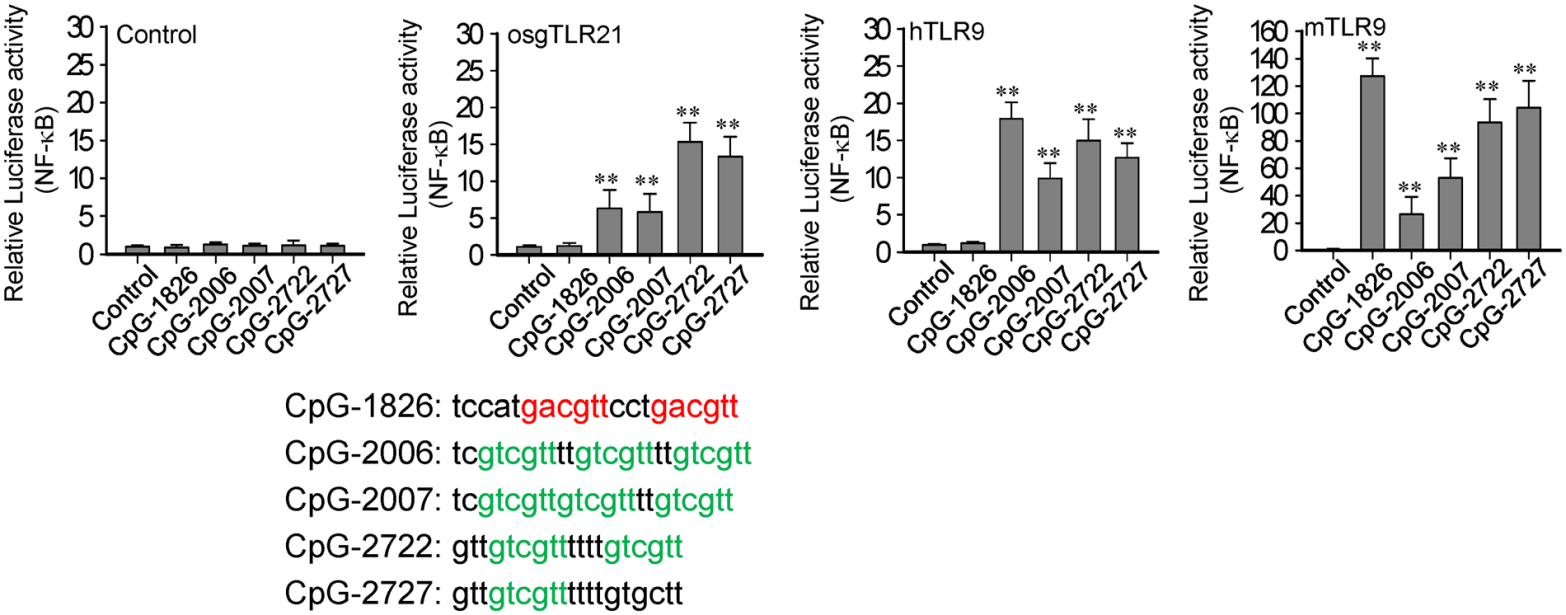
Activation of human and mouse toll-like receptor (TLR) 9 s by the CpG-oligodeoxynucleotides (CpG-ODNs) developed for grouper TLR21s. Relative luciferase activities of human embryonic kidney (HEK) 293 cells co-transfected with a control vector and expression vector for orange-spotted grouper (osg, *E. coioides*) TLR21, and human (h) and mouse (m) TLR9s as indicated, along with a nuclear factor (NF)-κB controlled luciferase reporter gene, and treated with 0.3 µM of different CpG-ODNs as indicated for 7 h. Data represent means ± SD (n = 3 independent experiments). **P < 0.01 compared with the control. The sequences of the CpG-ODNs used in this study are shown under the figures.

We tested the activities of CpG-2722 and CpG-2727 on human (h) and mouse (m) TLR9s to determine whether they are specific to grouper TLR21s. Interestingly, in the hTLR9 activation assay, we found that the activities of CpG-2722 and CpG-2727 were as good as those of CpG-2006 and CpG-2007, which have been optimized for the activation of human cells. Furthermore, in the mTLR9 activation assay, although the activities of CpG-2722 and CpG-2727 were not as good as that of CpG-1826, which has been optimized for the activation of mouse cells^8,22–24^, both had better activities toward mTLR9 than CpG-2006 and CpG-2007 (Fig. 3).

### Induction of the immune response by CpG-2722 and CpG-2727 in grouper, human, and mouse cells

**Figure 4:**
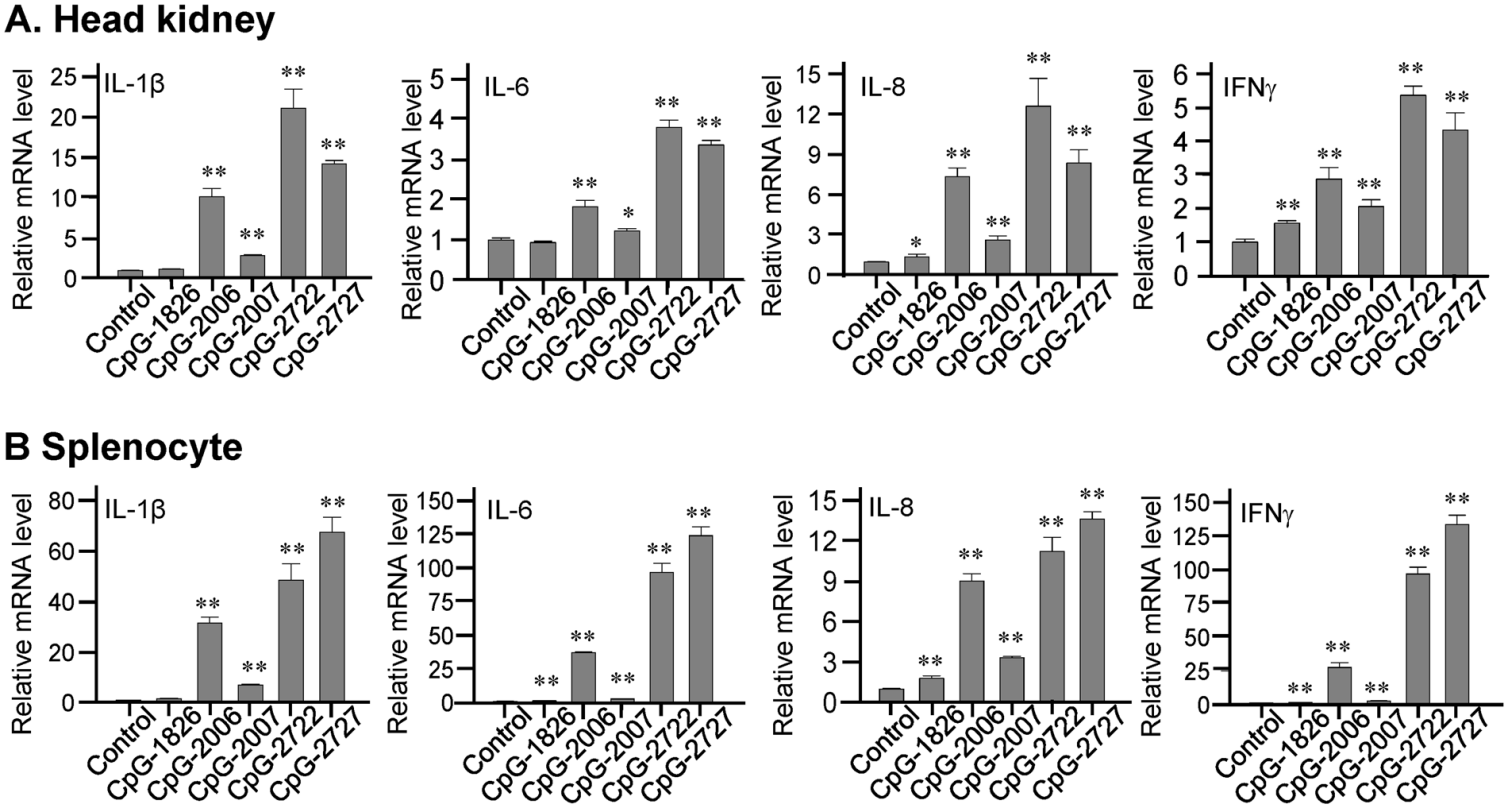
Induction of cytokine expression in orange-spotted grouper (*Epinephelus coioides*) cells by CpG-oligodeoxynucleotides (CpG-ODNs) developed for grouper toll-like receptor (TLR) 21 s. Expression of the cytokines interleukin (IL)-1β, IL-6, and IL-8, and interferon (IFN)γ in (**A**) head kidney cells and (**B**) splenocytes treated with 0.3 µM of different CpG-ODNs as indicated for 4 h and analyzed with reverse transcription-quantitative polymerase chain reaction (RT-qPCR). Data represent means ± SD (n = 3 independent experiments). *P < 0.05, **P < 0.01 compared with the control. The primers used in this study are shown in Supplementary Table 1.

**Figure 5:**
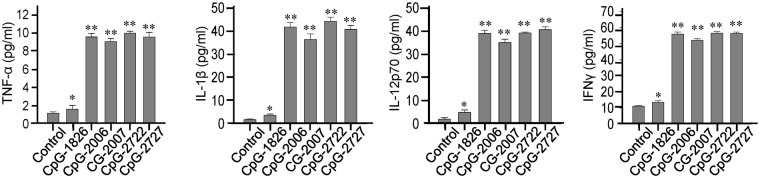
Induction of cytokine production in human cells by CpG-oligodeoxynucleotides (CpG-ODNs) developed for grouper (*Epinephelus* spp.) toll-like receptor (TLR) 21 s. Expression of the cytokines tumor necrosis factor (TNF)-α, interleukin (IL)-1β, IL-12p70, and interferon (IFN)γ by human peripheral blood mononuclear cells (PBMCs) treated with 0.3 µM of different CpG-ODNs as indicated for 24 h and analyzed with an enzyme-linked immunosorbent assay (ELISA). Data represent means ± SD (n = 3 independent experiments). *P < 0.05, **P < 0.01 compared with the control.

**Figure 6:**
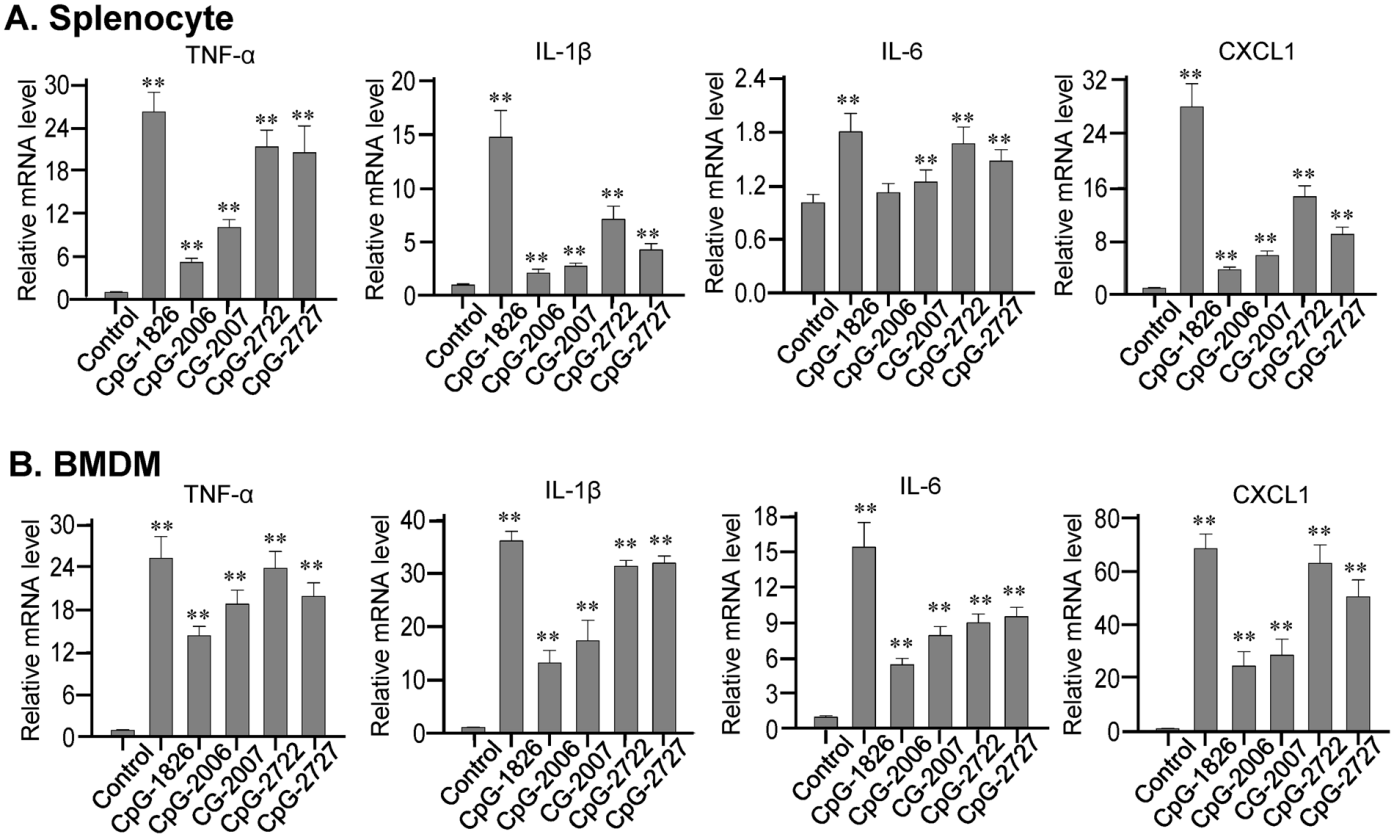
Induction of cytokine production in mouse cells by CpG-oligodeoxynucleotides (CpG-ODNs) developed for grouper (*Epinephelus* spp.) toll-like receptor (TLR) 21 s. Expression of the cytokines tumor necrosis factor (TNF)-α, interleukin (IL)-1β, IL-6, and CXCL1 by (**A**) mouse splecocytes and (**B**) bone marrow-derived macrophages (BMDMs) treated with 0.3 µM of the indicated CpG-ODNs for 4 h and analyzed with reverse transcription-quantitative polymerase chain reaction (RT-qPCR). Data represent means ± SD (n = 3 independent experiments). **P < 0.01 compared with the control. The primers used in this study are shown in Supplementalry Table 1.

We further investigated the effects of CpG-2722 and CpG-2727 on the induction of cytokine productions in grouper, human, and mouse cells to compare their activities on these cells with that showed on the cell-based activation assays in Fig. 3. Head kidney cells and splenocytes were purified from orange-spotted groupers, treated with different CpG-ODNs for determining the induction of the cytokines IL-1β, IL-6 IL-8, and IFNγ. Consistent with results obtained from the cell based assays, we found that CpG-2722 and CpG-2727 had a greater effect on the activation of cytokine production in orange-spotted grouper cells than CpG-1826, CpG-2006, and CpG-2007 (Fig. 4). An ELISA analysis showed that CpG-2722 and CpG-2727 were as potent as CpG-2006 and CpG-2007 in inducing cytokine production in human PBMCs, whereas CpG-1826 had weak activity on these cells (Fig. 5). Furthermore, CpG-2722 and CpG-2727 were more potent than the CpG-2006 and 2007 in inducing cytokine expression in mouse splenocytes and BMDMs (Fig. 6)

Overall, these findings suggest that the grouper TLR21s were activated by CpG-ODNs containing the GTCGTT motif but not by those containing the GACGTT motif. The two developed CpG-ODNs, CpG-2722 and CpG-2727, which contained 19 phosphorothiolated deoxynucleotides with two copies and one copy of GTCGTT motif respectively had better activity on grouper TLR21s than currently developed CpG-ODNs. These two CpG-ODNs also effectively activate immune responses in human and mouse cells.

## Discussion

TLR9 and TLR21 are the cellular receptors for CpG-ODNs. TLR9 is expressed in both mammalian and fish species, and has been better studied in terms of the structural requirements for strong CpG-ODN activation and species-specific activation. CpG-ODNs that have been optimized for humans are currently being investigated for their application as vaccine adjuvants, as well as immunotherapies for allergies, infectious diseases, and cancers^2,15,16,52^. Similarly, CpG-ODNs have also been shown to exert potent immunostimulatory activities in chicken, duck, and fish, which contain TLR21^11–14,35,38^, suggesting potential veterinary uses of CpG-ODNs as immune modulators and vaccine adjuvants. However, few studies have investigated the structural requirements for CpG-ODN to activate TLR21, and it remains unclear whether there is a common structure that will activate both TLR9 and TLR21. Therefore, in this study, we developed CpG-ODNs for strong activation of TLR21s in grouper, which are considered an important aquaculture species in Asia, due to their rapid growth and good selling price^41–43^, and investigated the interaction between CpG-ODNs, grouper TLR21s, and human and mouse TLR9s.

We found that CpG-ODNs that contained the GTCGTT motif (CpG-2006 and CpG-2007) activated grouper TLR21s, whereas those that contained the GACGTT and AACGTT motifs (CpG-1826, CpG-2000, and HC4040) did not. Similarly, it has previously been shown that zebTLR9s broadly recognize CpG-ODNs with different CpG-hexamer motifs, with those containing the GACGTT or AACGTT motifs having better activity toward TLR9, and those with the GTCGTT motif having better activity toward zebTLR21^40^. Thus, the grouper TLR21s have a similar CpG-hexamer recognition profile to zebTLR21.

In addition, we successfully developed two CpG-ODNs that have better activities toward grouper TLR21s than CpG-2006 and CpG-2007. CpG-2722 has a length of 19 deoxynucleotides and contains two GTCGTT motifs with four spacing nucleotides between them, while CpG-2727 is a modification of CpG-2722 in which the CpG-dideoxynucleotides in the 3′-GTCGTT motif has been reversed. Therefore, the good activity of CpG-2727 suggests that one copy of the GTCGTT motif is sufficient for strong activation of grouper TLR21s. In a previous study that developed CpG-ODNs for strong activation of rabbit TLR9, it was found that the length of a CpG-ODN also affects its activity^25^. Thus, the fact that CpG-2722 and CpG-2727 contain the same type of CpG-hexamer as CpG-2006 and CpG-2007, but have different lengths and numbers of the CpG-hexamer suggests that the key structural elements for designing CpG-ODNs to strongly activate TLR21 are the same as those for TLR9.

We further investigated the activities of CpG-2722 and CpG-2727 in cells isolated from the head kidneys and spleens of orange-spotted groupers, which should contain both TLR9 and TLR21. This showed that although the activation profile was not entirely the same as for grouper TLR21s, CpG-2722 and CpG-2727 exhibited a greater effect on cytokine production in these cells than that by CpG-2006, CpG-2007, and CpG-1826. Thus, the developed CpG-2722 and CpG-2727 may also have good activity toward grouper TLR9, and it appears that both TLR9 and TLR21 cooperatively mediate the immunostimulatory activities of CpG-ODNs in groupers, as previously demonstrated for zebTLR9 and zebTLR21 by CpG-ODNs with the GTCGTT motif^40^. Alternatively, this may suggest that TLR21 is the major receptor for CpG-ODNs in grouper cells.

Human and mouse TLR9s have been shown to have species-specific ligand recognition properties, with human TLR9 being strongly activated by CpG-2006 and CpG-2007, but weakly activated by CpG-1826, and mouse TLR9 being preferentially activated by CpG-1826. Here, we found that the activities of CpG-2722 and CpG-2727 were as strong as those of CpG-2006 and CpG-2007 in terms of the activation of human TLR9 and cytokine production in human PBMCs. Furthermore, these two CpG-ODNs also had better effects than CpG-2006 and CpG-2007 on the activation of mouse TLR9 and cytokine production in mouse cells. This suggests that these two developed CpG-ODNs contain structure composed by their nucleotide length and number of GTCGTT motif for better overriding the specific-specific ligand recognition properties of human and mouse TLR9s. In addition, they share a common structure for the activation of TLR9s and TLR21s from different species. Although the interactions of CpG-ODN with TLR9 and TLR21 from different species including domestic animals, birds and fish have not yet fully investigated, several reports have demonstrated the immunological activities of CpG-ODNs toward cells from these species^12–18^. Thus, these two CpG-ODNs have potential to be commonly used as immunomodulatory or vaccine adjuvants in a range of species.

## Methods

### Reagents and antibodies

CpG-oligodeoxynucleotides (CpG-ODNs) were purchased from Invitrogen (Carlsbad, CA, USA) or Genomics BioSci & Tech (New Taipei, Taiwan). Anti-FLAG® antibody, Tricaine, and all of the chemicals were purchased from Sigma-Aldrich (St. Louis, MO, USA), while anti-actin antibody was purchased from Santa Cruz Biotech Inc. (Dallas, TX, USA). Enzyme-linked immunosorbent assay (ELISA) kits for the detection of human cytokines were purchased from eBioscience (San Diego, CA, USA), and luciferase assay reagents were purchased from Promega (Madison, WI, USA).

### Animal studies

All studies performed with fish and mice were conducted in accordance with the protocols approved by the Institutional Animal Care and Use Committee of the National Health Research Institutes (approval number: NHRI-IACUC-103050A and NHRI-IACUC-104140).

### Isolation of head kidney cells and splenocytes from groupers

We anesthetized 2–3 month-old male and female orange-spotted groupers and giant groupers (obtained from Merit Ocean Biotech, Tainan, Taiwan) in water containing 0.2 g/l Tricaine, and aseptically removed the head kidneys and spleens. These organs were then gently minced by scissors and pressed with the head of the syringe plunger to pass through a 70-μm strainer with a homogenization buffer (standard Hank’s balanced salt solution (sHBSS) supplemented with 15 mM HEPES, 10% fetal bovine serum (FBS), 1 × Antibiotic-Antimycotic (Gibco, Carlsbad, CA, USA), and 50 U/ml heparin). Similar with an established procedure^53,54^, we then placed 1 ml of the homogenized tissue suspension into 8 ml distilled water and 1 ml 10 × phosphate-buffered saline (PBS). Following the removal of gross debris via drawing by a pipette, the cell suspension was centrifuged at 400 g at room temperature and washed twice with 1 × PBS. The cell pellet was then resuspended in Leibovitz’s L-15 cell culture medium (Gibco, Carlsbad, CA, USA).

### Cell preparation and culture

We collected mouse bone marrow-derived macrophages (BMDMs) and splenocytes from 6–8 week-old male and female C57BL/6 mice which were bred in the animal facility of NHRI. To generate the BMDM, mouse bone marrow cells from mouse tibias and femurs were cultured in complete Dulbecco’s Modified Eagle Medium (DMEM) with 30% L929 conditioned medium for 5 days, followed by DMEM medium with 10% FBS. Mouse splenocytes were cultured in RPMI1640 medium with 10% FBS. Human peripheral blood mononuclear cells (PBMCs) were prepared in accordance with the protocol approved by the Institutional Review Board (IRB) of National Yang-Ming University (IRB #YM102026E). Written informed consent was obtained from donors before entering the study protocol. These PBMCs were cultured in RPMI1640 medium with 10% FBS.

### Molecular cloning of ggTLR21 cDNA

Total RNAs were purified from grouper splenocytes using TRIzol, and first-strand cDNA libraries were then prepared using the SuperScript® III First-Strand Synthesis System (Invitrogen, Carlsbad, CA, USA), as previously described^40^. To clone ggTLR21 cDNA, a pair of forward and reverse primers (5′-gaacagattcctgtaccatgttcatc-3′ and 5′-gcttgtatgaattgtcacactgcac-3′) were designed based on the sequences of the 5′- and 3′-untranslated regions of osgTLR21 (GenBank: GU198366.2). A ggTLR21 cDNA containing both the 5′-and 3′-untranslated regions and the complete coding region was cloned from the prepared giant grouper spleen first-strand cDNA library using polymerase chain reaction (PCR) amplification with a Phusion® High-Fidelity DNA Polymerase (New England Biolabs, Ipswich, MA), following the conditions recommended by the manufacturer.

### Bioinformatic analysis

Multiple alignment of the amino acid sequences of the TLR21s was performed using ClustalW2 (http://www.ebi.ac.uk/Tools/msa/clustalw2/). The phylogenetic tree was created by the neighbor joining method. The structural model of the TLR21 protein was predicted with SWISS MODEL (http://www.swissmodel.expasy.org/), using TLR13 as a template.

### Expression vectors for TLR21s and TLR9s

We generated the expression constructs for osgTLR21s and ggTLR21 through PCR amplification of the corresponding protein-coding regions from the first-strand cDNA library derived from the generated orange spotted and giant grouper spleen first-strand cDNA libraries. We designed the forward and reverse primers based on the 5′-and 3′-end cDNA sequences for the coding region of osgTLR21 (GU198366.2, 5′-atggcgagtctaacttatcagctg -3′, 5′-ttatggaagcaagtaatagttttcatc-3′) and ggTLR21 (KM024068, 5′-atggcgagtctaacttatcagctg-3′ and 5′-ttatggaagcaagtaatagttttcatc-3′). PCR reactions were performed using a Phusion® High-Fidelity DNA Polymerase (New England Biolabs, Ipswich, MA) following the conditions recommended by the manufacturer. The amplified coding regions were subcloned the amplified DNA fragments into a PEF6 vector in frame with a FLAG tag at their C-terminal ends. The expression vectors for human and mouse TLR9 were generated following previously described methods^55^.

### TLR21 and TLR9 activation assays

Human embryonic kidney (HEK) 293 cells were grown in DMEM supplemented with 10% fetal bovine serum, and TLR21 and TLR9 activation assays were performed as previously described^40^. Briefly, the cells were plated on 24-well plates and allowed to adhere overnight. These cells were co-transfected using polyethylenimine (Sigma-Aldrich, St. Louis, MO, USA) with TLR921 or TLR9 expression vector, β-galactosidase plasmid, and an NF-κB-driven luciferase reporter plasmid. On the next day, these transfected cells were treated with different concentrations of various CpG-ODN as indicated for 7 h. The cells were lysed, and luciferase activity in each sample was determined. Relative luciferase activities were calculated as fold induction compared with an unstimulated control. The data are expressed as mean ± SD (n = 3).

### Reverse transcription-quantitative PCR (RT-qPCR) analysis of gene expression

Cells were treated with different CpG-ODNs for 4 h. We then purified the total RNA using TRIzol and performed reverse transcription using the SuperScript III First-Strand Synthesis System (Invitrogen, Carlsbad, CA, USA). RT-qPCR was carried out using an ABI PRISM 7900HT Sequence Detection System and KAPA SYBR® fast qPCR kit (KK4605) for gene expression analysis. The expression of mRNA was normalized to β-actin. The primer sequences for this RT-qPCR analysis are listed in Supplementary Table 1.

### Sodium dodecyl sulfate polyacrylamide gel electrophoresis (SDS-PAGE) and immunoblot analysis

Cells were lysed with lysis buffer (100 mM NaCl, 50 mM Tris-Cl (pH 7.5), 0.5 mM ethylenediaminetetraacetic acid (EDTA), 1% nonyl phenoxypolyethoxyethanol (NP-40)) containing complete protease inhibitor cocktail (Roche Life Science, Indianapolis, IN, USA). Cell lysates were separated by SDS-PAGE and transferred onto polyvinylidene fluoride (PVDF) membranes. We first incubated the membranes with the indicated antibody and then with horseradish peroxidase (HRP)-conjugated secondary antibody, following which we visualized the immunoreactive bands using chemiluminescent HRP substrate (Immobilon Western; Millipore, Temecula, CA, USA) and the UVP BioSpectrum Imaging System.

### Measurement of mouse and human cytokine production

We treated PBMCs with different CpG-ODNs for 24 h, and then collected the culture supernatants for cytokine measurement. We measured the production of tumor necrosis factor (TNF)-α, interleukin (IL)-1β, IL-12p70, and interferon (IFN)-γ using ELISA Kits (eBioscience, San Diego, CA, USA) according to the manufacturer’s instructions.

### Statistical analysis

All data are expressed as the mean ± SD of three independent experiments. Statistical analyses were performed using Student’s *t*-test with a significance level of *P* < 0.05.

### Data availability

We submitted the giant grouperTLR21 cDNA sequence to the GenBank database (accession number: KM024068).

## Electronic supplementary material

Supplementary information
