# Supplementary material for: CpG-oligodeoxynucleotides developed for grouper toll-like receptor (TLR) 21s effectively activate mouse and human TLR9s mediated immune responses
Source: Sci Rep. 2017 Dec 11;7:17297. doi: 10.1038/s41598-017-17609-2 (PMC5725558; doi:10.1038/s41598-017-17609-2)

Supplementary Information

CpG-oligodeoxynucleotides developed for grouper toll-like receptor (TLR) 21s effectively activate mouse and human TLR9s mediated immune responses

Da-Wei Yeh[1*], Chao-Yang Lai[1*], Yi-Ling Liu[1], Chih-Hao Lu[1,2], Ping-Hui Tseng[3], Chiou-Hwa Yuh[4], Guann-Yi Yu[5], Shih-Jen Liu[5], Chih-Hsiang Leng[5], & Tsung-Hsien Chuang[1,6,]

[1] Immunology Research Center, National Health Research Institutes, Miaoli, Taiwan
[2] Department of Life Sciences, National Central University, Taoyuan, Taiwan
[3] Institute of Biochemistry and Molecular Biology, National Yang-Ming University, Taipei, Taiwan
[4] Institute of Molecular and Genomic Medicine, National Health Research Institutes, Miaoli, Taiwan
[5] National Institute of Infectious Diseases and Vaccinology, National Health Research Institutes, Miaoli, Taiwan.
[6] Program in Environmental and Occupational Medicine, Kaohsiung Medical University Kaohsiung, Taiwan

*These authors contributed equally to this work.
Correspondence and requests for materials should be addressed to T.-H.C. (email: thchuang@nhri.org.tw)

Supplementary Table 1. Nucleotide sequences of the primers used for reverse transcription-quantitative polymerase chain reaction (RT-qPCR).

Supplementary Figure 1. Alignment of toll-like receptor (TLR) 21 protein sequences for orange-spotted grouper (*Epinephelus coioides*; osgTLR21A and osgTLR21B) and giant grouper (*E. lanceolatus*; ggTLR21). LRR, leucine-rich repeat; LRR-CT, C-terminal LRR; TM, transmembrane domain; Box1–3, box 1–3 in the cytosolic Toll/interleukin receptor (TIR) domain. Amino acids are color-coded to indicate their chemical properties: blue, acidic; pink, basic; green, hydroxyl/amine/basic/Q; red, hydrophobic (including aliphatic Y). Asterisk, identical residues; single dot, conservative substitutions; two dots, highly conservative substitutions.

Supplementary Figure 2. Phylogenetic analysis of grouper (*Epinephelus* spp.) toll-like receptor (TLR) 21s and zebrafish (zeb, *Danio rerio*) TLRs. Numbers in the left column represent the percentage protein identities of each TLR to giant grouper (gg, *E. lanceolatus*) TLR21. The GenBank accession numbers of these TLR protein sequences are listed in the right column.

Supplementary Figure 3. Phylogenetic analysis of fish toll-like receptor (TLR) 21s. Numbers in the left column represent the percentage protein identities of each fish TLR21 to giant grouper (gg, *E. lanceolatus*) TLR21. The GenBank accession numbers of these TLR protein sequences are listed in the right column.

Supplementary Figure 4. Full length immunoblots of figure 1D. Total cell lysates were collected, separated by SDS-PAGE, blotted to polyvinylidene fluoride (PVDF) membrane, and probed with primary and secondary antibodies as described in the methods section.

Supplementary table 1

Grouper Primers used in RT-qPCR

β-actin	forward 5'-GACATGGTGCGGTTTCTCTT-3'
	reverse 5'-GCCTCTGCTGTGCTGATGTA-3'
IL-1β	forward 5'-GACATGGTGCGGTTTCTCTT-3'
	reverse 5'-GCCTCTGCTGTGCTGATGTA-3'
IL-6	forward 5'-CCTGAAGGACCTCGACAATC-3'
	reverse 5'-TCCTGACAGCCAGACTTCCT-3'
IL-8	forward 5'-GAGCTGCACTGTCGCTGTAT-3'
	reverse 5'-TGTTGGCCATGATCCTGTTA-3'
IFNγ	forward 5'-GACCACCAAGATGGAGGCTA-3'
	reverse 5'-TACCGGTGTTTCCTCAGGTC-3'

Mouse Primers used in RT-qPCR

gapdh	forward 5'-ACCCAGAAGACTGTGGATGG-3'
	reverse 5'-CACATTGGGGGTAGGAACAC-3'
tnf-α	forward 5'-GGATCTGGCGCTACTCAGAC-3'
	reverse 5'-TCCGATAGCTGGTTGGTTTC-3'
il-1β	forward 5'-CAGGCAGGCAGTATCACTCA-3'
	reverse 5'-AGCTCATATGGGTCCGACAG-3'
il-6	forward 5'-AGTTGCCTTCTTGGGACTGA-3'
	reverse 5'-TCCACGATTTCCCAGAGAAC-3'
cxcl1	forward 5'-GCTGGGATTCACCTCAAGAA-3'
	reverse 5'-CTTGGGGACACCTTTTAGCA-3'

Suplementary figure 1

```
                                             LRR1
osgTLR21A  MASLTYQLLSVTLALCAVHLISGYSFRNCIEDPYPYGQSFKCILRKESNLSALIGDLPQT  60
osgTLR21B  MASLTYQLLSVTLALCAVHLISGYSFRNCIEDPYPYGQSFKCILRKESNLSALIGDLPQT  60
ggTLR21    MASLTYQLLSVTLALCAVHLISGYSFRNCIEDPYPYGQSFKCILRKESNLSAVIGDLPQT  60
           ****************************************************:*******

               LRR2               LRR3                    LRR4
osgTLR21A  AVNLTITINPVWHIPSMSFVNVTNLEYLRLDHNYLRKIDPFAFQNLTRLKSLNVSSNNIS  120
osgTLR21B  AVNLTITINPVWHIPSMSFVNVTNLEYLRLDHNYLRKIDPFAFQNLTRLKSLNVSSNNIS  120
ggTLR21    AVNLTITINPVWHIPSMSFVNVTNLEYLRLDHNYLRKIDPFAFQNLTRLKSLNVSSNNIS  120
           ************************************************************

                                 LRR5
osgTLR21A  QLNPYVFKDLHNLTYLSLTNNSLKQLPVGIFSTLLNLDTLIMRQNLLTNFSGIAESVSHL  180
osgTLR21B  QLNPYVFKDLHNLTYLSLTNNSLKQLPVGIFSTLLNLDTLIMRQNLLTNFSGIAESVSHL  180
ggTLR21    QLNPYVFKDLHNLTYLSLTNNSLKQLPVGIFSTLLNLDTLIMRQNFLTNFSGIAESVSHL  180
           *********************************************:**************

                 LRR6                      LRR7
osgTLR21A  PKLRVLDLCFNNLTNLKHSNASLPKSLTTLYICRNNLLTLGCHQSFLGFIQLLDLSYNPR  240
osgTLR21B  PKLRVLDLCFNNLTNLKHSNASLPKSLTTLYICRNNLLTLGCHQSFLGFIQLLDLSYNPR  240
ggTLR21    PKLRVLDLCFNNLTNLKHSNASLPKSLTTLYICRNNLLTLGCHQSFLGFIQLLDLSYNPR  240
           ************************************************************

                 LRR8                         LRR9
osgTLR21A  LPTMAFQGVDLSHINYLRLRSTSVKVVDFLNISNVNAGSVDFSGMGLKNDILLTELCTSL  300
osgTLR21B  LPTMAFQGVDLSHINYLRLRSTSVKVVEFLNISNVNAGSVDFSGMGLKNDILLTELCTSL  300
ggTLR21    LPTMAFQGVDLSHINYLRLRSTSVKVVEFLNISNVNAGSVDFSGMGLKNDTLLTELCTSL  300
           ***************************:********************** *********

                     LRR10                 LRR11
osgTLR21A  KGKVKSIKRMGLSNNGIKNLTNNALQYCPTITGSLDLSRNNLKSTGCFKFLDKHTQIKSL  360
osgTLR21B  KGKVKSIKRMGLSNNGIKNLTNNALQYCPTITGSLDLSRNNLKSTGCFKFLDKHTQIKSL  360
ggTLR21    KGKVKSIKRMGLSNNGIKNLTNYALQYCPTITGSLDLSRNNLKSTGCFKFLDKHTQIKSL  360
           ********************** *************************************

               LRR12           LRR13                          LRR14
osgTLR21A  NAEHNHITSLQSCKTQNMVYLNHLEELSYRYNRILSVNAYAFSHTPNIKTLKLNINTISF  420
osgTLR21B  NAEHNHITSLQSCKTQNMVYLNHLEELSYRYNRILSVNAYAFSHTPNIKTLKLNINTISF  420
ggTLR21    NAEHNHITSLQSCKTENMVYLNHLEELSYRYNRILSVNAYAFSHTPNIKTLKLNINTISF  420
           ***************:********************************************

                           LRR15                  LRR16
osgTLR21A  LHRKALKGLKSLEMLRLDNNLLTDLFNDTFEDNVNLQTLNLRNNRIAVIFNGTFLSLRNL  480
osgTLR21B  LHRKALKGLKSLEMLRLDNNLLTDLFNDTFEDNVNLQTLNLRNNRIAVIFNGTFLSLRNL  480
ggTLR21    LHRKALKGLKSLEMLRLDNNLLTDLFNDTFEDNVNLQTLNLRNNRIAVIFNGTFLSLRNL  480
           ************************************************************

                 LRR17                        LRR18
osgTLR21A  TTLDLGGNKITHFEQSGLDGLTSLSKFYLDGNNLKEIDTSLYRVFQDTLTVLDLKSNQIY  540
osgTLR21B  TTLDLGGNKITHFEQSGLDGLTSLSKFYLDGNNLKEIDTSLYRVFQDTLTVLDLKSNQIY  540
ggTLR21    TTLDLGGNKITHFEQSGLDGLTSLSKFYLDGNNLKEIDTSLYRVFQDTLTVLDLKSNQIY  540
           ************************************************************

                               LRR19                    LRR20
osgTLR21A  FFHKVTSSPFMNLSKLTDLKLDGQRPHGLSVLPQNFFRGLHSLKSLYLTNNNIYYLAPDA  600
osgTLR21B  FFHKVTSSPFMNLSKLTDLKLDGQRPHGLSVLPQNFFRGLHSLKSLYLTNNNIYYLAPDA  600
ggTLR21    FLHKVTSSPFMNLSKLSDLKLDGQRPHGLSVLPQNFFRGLHSLKSLYLANNNIYYLAPDA  600
           *:**************:*******************************:***********

                      LRR21                    LRR22
osgTLR21A  FDDLTNLTFLSLEGCCVGVAQLQPGIFKNLRNLNILSMENMGIQTFSKEVFGNLTKLHKL  660
osgTLR21B  FDDLTNLTFLSLEGCCVGVAQLQPGIFKNLRNLNILSMENMGIQTFSKEVFGNLTKLHKL  660
ggTLR21    FDDLTNLTFLSLESCCVGVAQLQPGIFKNLRNLNILSMENMGIQTFSKEVFGNLTKLHKL  660
           *************.**********************************************

               LRR23                                      LRR CT
osgTLR21A  QLNRNVMQSIHYEILDSLPELHYLDLRDTPLSCTCKNHLLQNWTLHNRRVQVVYLYNMKC  720
osgTLR21B  QLNRNVMQSIHYEILDSLPELHYLDLRDTPLSCTCKNHLLQNWTLHNRRVQVVYLYNMKC  720
ggTLR21    QLNRNVMQSIHFEILDSLPELHYLDIRNTPLSCTCKNHLLQNWTLHNRRVQVVYLYNMKC  720
           ***********:*************:*:********************************

                                                     TM
osgTLR21A  QDDNQHNFYNFKTNVCYIDLGEYLFLSTAIVIFLFTVTPLLYVKLYWKMKYGYYVFRSWF  780
osgTLR21B  QDDNQHNFYNFKTNVCYIDLGEYLFLSTAIVIFLFTVTPLLYVKLYWKMKYGYYVFRSWF  780
ggTLR21    PDDNQHNFYNFKTNVCYIDLGEYLFLSTAIVIFLFTVTPLLYVKLYWKMKYNYYVFRSWF  780
            **************************************************.********

                         Box 1                             Box2
osgTLR21A  SDQWRRLREQEENCKYDAFISYNSSDEQWVIEQLMPNLEGNGSSFKLCLHHRDFELGRDI  840
osgTLR21B  SDQWRRLREQEENCKYDAFISYNSSDEQWVIEQLMPNLEGNGSSFKLCLHHRDFELGRDI  840
ggTLR21    SDQWRRLREQEENCKYDAFISYNTSDEQWVIEQLMPNLEGNGSSFKLCLHHRDFELGRDI  840
           ***********************:************************************

osgTLR21A  VDNIVSAVYSSRKTICVVSRNFLTSEWCSLEIQLASYRLFDEHRDVLLLVFLEPISERQL  900
osgTLR21B  VDNIVSAVYSSRKTICVVSRNFLTSEWCSLEIQLASYRLFDEHRDVLLLVFLEPISERQL  900
ggTLR21    VDNIVSAVYSSRKTICVVSRNFLTSEWCSLEIQLASYRLFDEHRDVLLLVFLEPISERQL  900
           ************************************************************

                                           Box3
osgTLR21A  SSYHRMRKVMLKKTYLQWPGSDCTNPTQAQELFWNQLRRAMRTGSRFETEQNCSEGREKE  960
osgTLR21B  SSYHRMRKVMLKKTYLQWPGSDCTNPTEAQELFWNQLRRAMRTGSRFETEQNCSEGREKE  960
ggTLR21    SSYHRMRKVMLKKTYLQWPGSDCTNPTQAQELFWNQLRRAMRTGSRFETEQNCSEGREKE  960
           ***************************:********************************

osgTLR21A  GRTEHVDARTADENYYLLP  979
osgTLR21B  GRTEHVDE----QNYYLLP  975
ggTLR21    GRTEHVDARTSDENYYLLP  979
           *******     :******
```

Supplementary figure 2

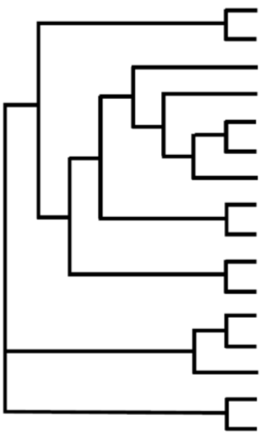

	% protein Identity to ggTLR21	GenBank Accession number
zebTLR3	20.16	NM_001013269
zebTLR5b	20.00	NM_001130595
zebTLR22	28.30	NM_001168275.2
zebTLR21	54.44	NM_001199335
osgTLR21A	98.26	GU198366.2
osgTLR21B	98.15	JF738114.1
ggTLR21	100.00	KM024068
zebTLR20f	23.51	XM_009304784.1
zebTLR13	21.96	XM_009304761.3
zebTLR4ba	20.13	NM_001131051
zebTLR4bb	20.56	NM_212813
zebTLR1	19.75	NM_001130593
zebTLR2	18.65	NM_212812
zebTLR18	17.45	NM_001089350
zebTLR8a	17.39	XM_001920559
zebTLR9	19.60	NM_001130594

Supplementary figure 3

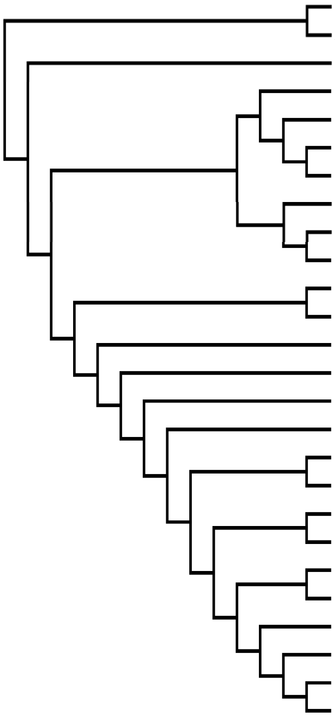

	% protein Identity to ggTLR21	GenBank or Ensembl Accession number
1: Anser_cygnoides_TLR21	43.58	KT735043.1
2: Gallus_gallus_TLR21	43.36	NM_001030558.1
3: Anguilla_japonica_TLR21	55.33	KU950320.1
4: Danio_rerio_TLR21	54.62	NM_001199335
5: Ctenopharyngodon_idella_TLR21	54.47	KC466564.1
6: Cyprinus_carpio_TLR21-1	55.31	LC150773.1
7: Cyprinus_carpio_TLR21-2	54.55	LC150774.1
8: Astyanax_mexicanus_TLR21	54.77	ENSAMXT00000025862.1
9: Clarias_batrachus_TLR21	56.59	KC907864.1
10: Ictulurus_punctatus_TLR21	56.33	NM_001200065.1
11: Coregonus_maraena_TLR21	62.45	LN610598.1
12: Salmo_salar_TLR21	63.07	HG514151.1
13: Gadus_morhua_TLR21	57.95	JX074771.1
14: Takifugu_rubripes_TLR21	65.18	NM_001032579.1
15: Oreochromis_niloticus_TLR21	67.83	KJ010824.1
16: Poecilia_formosa_TLR21	69.06	ENSPFOT00000020466.1
17: Gasterosteus_aculeatus_TLR21	71.66	ENSGACG00000009364.1
18: Notothenia_coriiceps_TLR21	72.50	KJ531979.1
19: Scophthalmus_maximus_TLR21	72.26	KU746965.1
20: Paralichthys_olivaceus_TLR21	73.15	JQ411238.1
21: Larimichthys_crocea_TLR21	71.38	KX928778.1
22: Miichthys_miiuy_TLR21	72.59	KR709255.1
23: Oplegnathus_fasciatus_TLR21	77.29	KJ004565.1
24: Epinephelus_lanceolatus_TLR21	100.00	KM024068
25: Epinephelus_coioides_TLR21A	98.26	GU198366.2
26: Epinephelus_coioides_TLR21B	98.15	JF738114.1

Supplementary figure 4

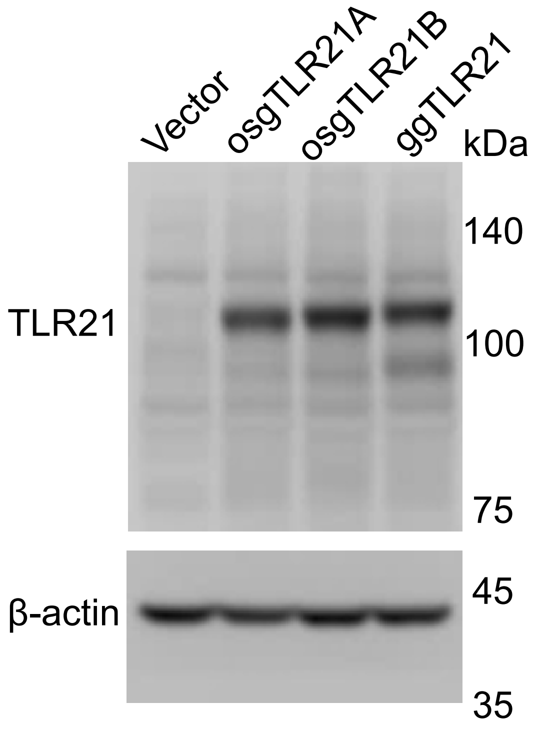

Supplement: Supplementary file 1 — Supplementary information [file 41598_2017_17609_MOESM1_ESM.pdf]
